# Observation of direction instability in a fiber ring laser

**DOI:** 10.1038/s41598-021-83042-1

**Published:** 2021-02-24

**Authors:** Muhammad Assad Arshad, Alexander Hartung, Arni Candra Pratiwi, Matthias Jäger

**Affiliations:** 1 Leibniz Institute of Photonic Technology, Albert-Einstein-Straße 9, 07745 Jena, Germany; 2 Faculty of Physics and Astronomy, Friedrich Schiller University, Max-Wien-Platz 1, 07743 Jena, Germany

**Keywords:** Optics and photonics, Lasers, LEDs and light sources, Optical materials and structures, Optical techniques, Other photonics

## Abstract

We report on the observation of a new phenomenon occurring in a fiber ring laser. This phenomenon is about the transition from an initially bidirectional emission of a reciprocal fiber ring laser to a unidirectional emission at a certain pump power threshold. In addition, the final direction is not predefined but appears to be randomly chosen every time the threshold is exceeded. Therefore, we term this new phenomenon direction instability. Furthermore, we provide a first discussion of how the instability threshold is influenced by the length and the loss of the cavity. We show that the threshold follows a power times length scaling, indicating a nonlinear origin.

## Introduction

Instability thresholds are a frequent phenomenon in lasers with a long and rich scientific history^1^. They are typically connected to a certain pump power threshold and require a thorough scientific understanding to develop mitigation strategies and to further extend the addressable parameter space of lasers in general.

An early example is the pump power threshold where the continuous-wave emission breaks up into pulses^2–5^. Due to the onset of temporal modulations this instability phenomenon is also termed *modulation instability* (MI). MI generally originates from small power perturbations in combination with nonlinear and dispersive interactions during light propagation. Nevertheless, there are still fundamental differences in MI occurring during cavityless nonlinear propagation^2,3^ compared to MI in a cavity^4,5^. A detailed understanding not only assisted the realization of high-power cw laser radiation but in addition enabled the generation of ultrashort very-high-repetition pulse trains^6^.

With the progress of laser technology to new highs of pump and laser power in combination with ever more sophisticated designs and layouts, new instability phenomena have occurred more recently. E.g., the so called *modal instability* refers to the phenomenon of the sudden transition of the modal output of a fiber laser from the fundamental mode to a higher order mode^7,8^. This results in a severe degradation of the beam quality. Cause-effect-relations have not been that obvious in this case and comprehensive investigations were required to develop appropriate mitigation strategies avoiding the thermally induced refractive index gratings which are the basis of this phenomenon^9,10^.

Instabilities are also often found in *random lasers*^11^. They constitute a special class of lasers where the feedback of the cavity is realized by a disordered scattering process. Random lasers have been realized in different media like powders and liquids and typically display quite complex and instable temporal, spectral, and spatial profiles. As an exception to this, random *fiber* lasers (RFL) have proven stable continuous-wave single-mode output under certain conditions^12,13^. RFLs were realized based on fundamentally different physical mechanisms. A major one relies on the disordered Rayleigh scattering in a kilometer-long telecom fiber in combination with stimulated Raman scattering to create the necessary feedback for lasing^14,15^. Another one relies on *population inversion dynamical gratings* in a cavity that is mainly constituted of the active fiber only^16,17^. Both mechanisms might have a relation to the phenomenon presented here, as will be discussed later.

In this manuscript we report about a new, recently^18^ observed instability phenomenon, here called *direction instability* (DI). DI is about the sudden transition from a bidirectional to a unidirectional emission of arbitrary direction, occurring in a fiber laser. The next section details the ring cavity layout we use, which is specifically designed to promote nonlinear effects. The third section introduces the general circumstances and properties of DI, which apply independent of a specific cavity layout. We demonstrate the transition from an even power distribution in both directions to essentially a one-way distribution. In the fourth section we discuss the impact of cavity variations on the DI threshold, specifically cavity length and loss, and reveal a power times length scaling indicating the nonlinear DI origin. The fifth section focuses on some statistical properties like the properties of switching to a clockwise or counterclockwise final direction. Finally, the manuscript is summarized and concluded in the last section.

In contrast to known laser instabilities DI has the potential to be of some use. A scientific understanding not only enables mitigation strategies in case you want to maintain bidirectional emission but also paves the way to reduce the requirements, e.g. the currently kilometer-long cavity length, for an easier access to this unique phenomenon.

From an application perspective, DI is a promising extension to the existing portfolio of unidirectionality approaches. Existing options of enforced unidirectionality all have their respective drawbacks like increased complexity, reduced power handling capability, or limited wavelength flexibility. These restrictions are removed by the new form of unidirectionality presented here, where no additional components are required, albeit new ones might apply. In addition, DI is an option where others are not available. Especially in new and emerging wavelength regions, e.g. the mid IR, components required to enforce unidirectionality like isolators, fused fiber couplers, tapers, polarization maintaining or polarizing fibers are not readily available. Getting rid of the need for these components by using DI as investigated here has the potential to accelerate the development and test of new laser systems in emerging spectral regions.

## Experimental setup

**Figure 1:**
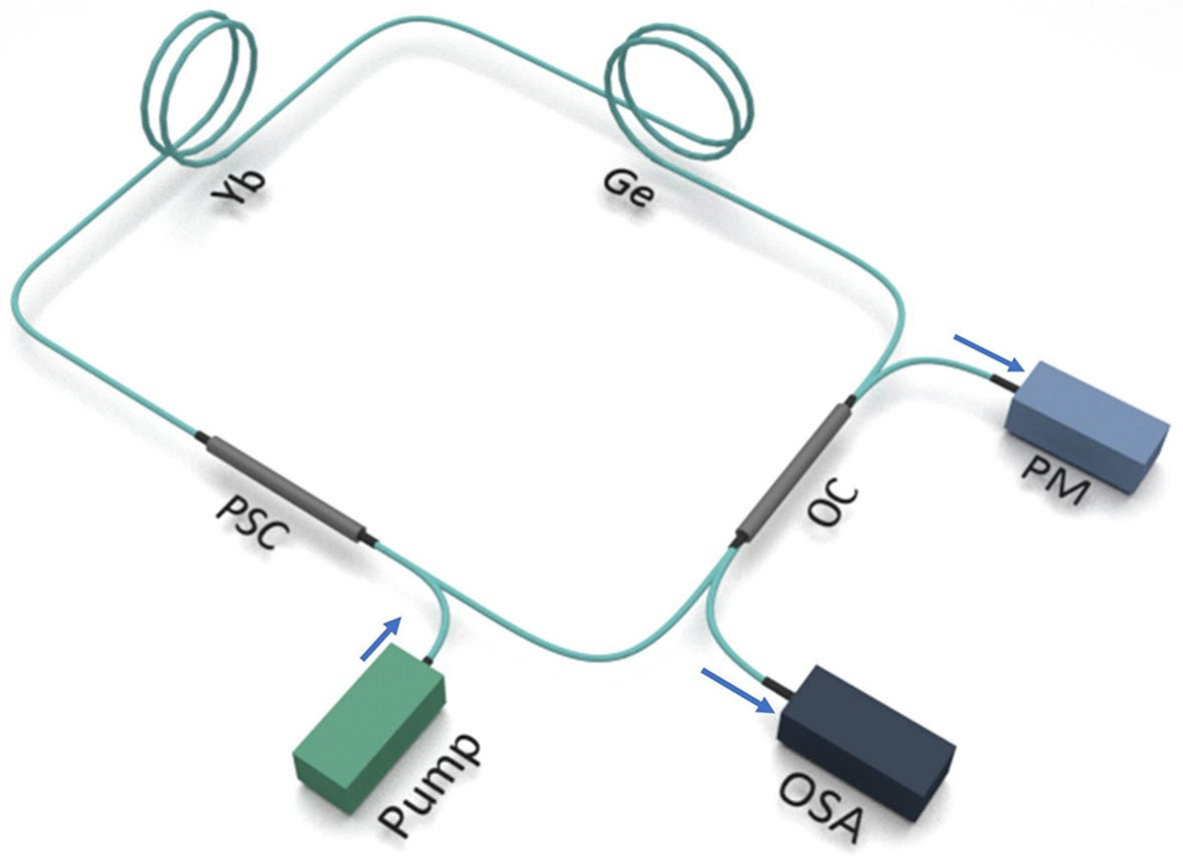
General scheme of the all-fiber ring laser. The setup consists of a pump diode (pump), pump signal combiner (PSC), an active Yb-doped fiber (Yb), a passive Ge-doped fiber (Ge), and an output coupler (OC). The output signal is monitored by an optical spectrum analyzer (OSA) and a power meter (PM).

The setup in use consists of an all-fiber ring cavity^18^ as shown in Fig. 1 and is constituted of commercially available components only. The main intention of this simple setup is to effectively trigger nonlinear effects. The cavity is pumped by a pump diode emitting at a wavelength of 976 nm up to a maximum power of 37 W. The pump power enters the ring cavity by a pump signal combiner and reaches a 6 m long cladding-pumped Yb-doped active fiber section. Here, without any wavelength selective filter applied, a laser signal at around 1100 nm is emitted. The active fiber is followed by a long (~ km) passive fiber (Corning HI-1060). Finally, an output tap coupler with a very low nominal coupling ratio of 0.01% at 1060 nm feeds most of the signal back to the pump signal combiner where it passes through the ring again, and a small part of the signal out of the cavity. As no optical isolator is included in the ring cavity clockwise and counterclockwise signals can propagate. To minimize roundtrip losses similar mode field diameters in the range of 6.1 µm to 6.6 µm and only single mode components are selected. All components are non-polarization maintaining.

Figure 1 displays a general layout. Different layouts with respect to length and loss of the passive fiber were investigated. Below is an explanation of the general behavior independent of the passive fiber length and loss. The impact of these specific parameters is explained thereafter.

### General properties

**Figure 2:**
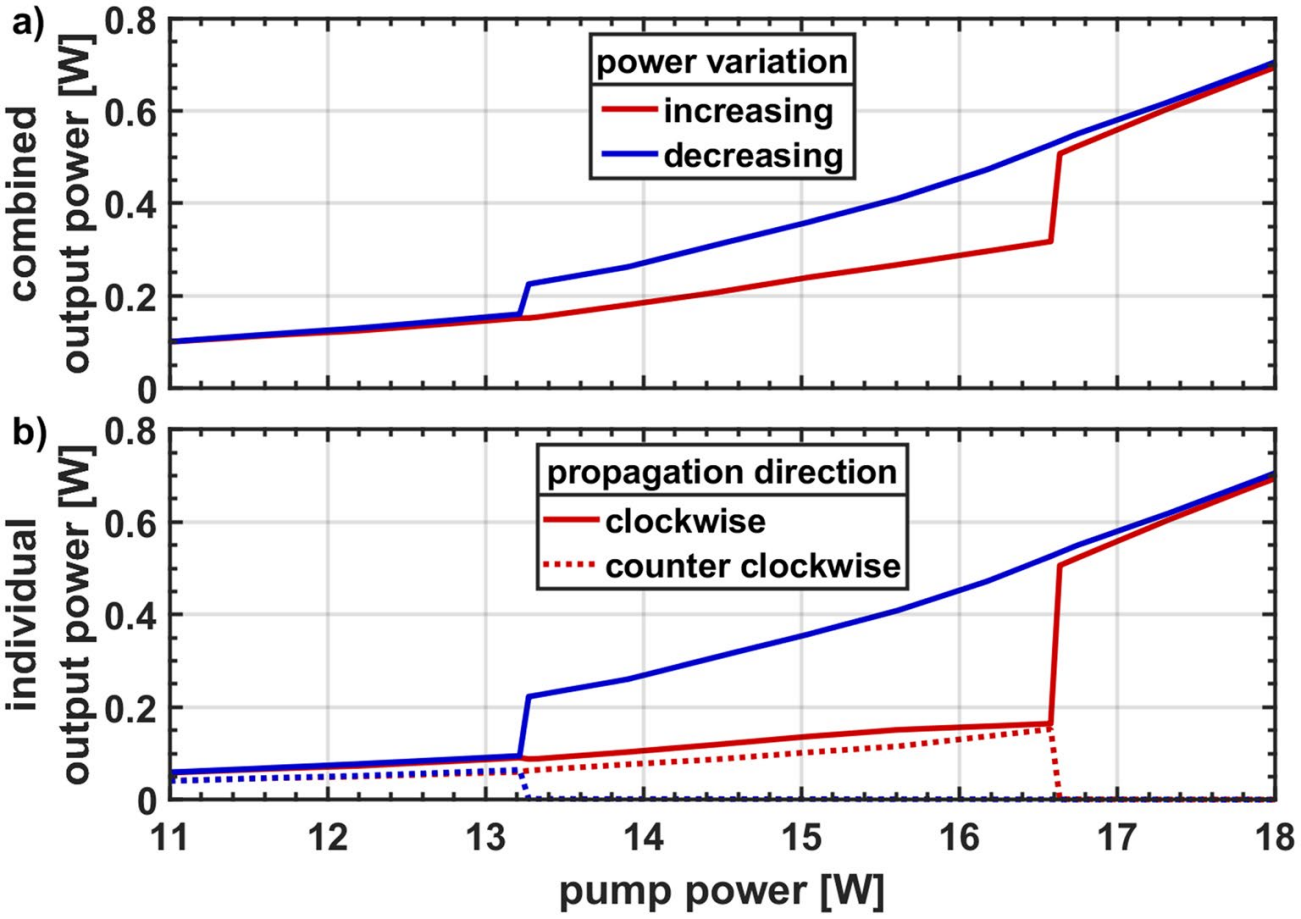
General evolution of (**a**) the combined output power and (**b**) the directional output powers demonstrating a bistability and unidirectional lasing behavior.

The two outputs of the ring cavity are characterized both in terms of the output power and spectrum. The trends of output powers are shown in Fig. 2, where the combined power from the two outputs is plotted in Fig. 2a) and individual powers are plotted in Fig. 2b). The combined output power is obtained by adding the individual powers from the two directions. The two directions were measured simultaneously with identical power meters.

Figure 2a shows that the combined output power steadily increases with increasing pump power up till 16.5 W. At this point a step is observed. We name this point the upper switching point. It happens far above the laser threshold, located approx. at 1 W. The pump power is further increased till 18 W and then subsequently decreased in a similar manner. The higher output power trend subsists far below the upper switching point till the pump power of 13.3 W where a sudden drop in output power is observed and the value falls to similar levels observed while the pump power was increased. We refer to this point as the lower switching point. The full behavior depicts a hysteresis region between the two switching points, showing the existence of optical bistability.

Figure 2b reveals a similar behavior for the individual output powers. Here we observe that the power from the two counter propagating directions start with similar values and steadily increase in a similar manner till the upper switching point. At this point the output power from the clockwise directions suddenly enhances in magnitude (by roughly three times) while the power from the counterclockwise direction falls significantly to just a few mill watts. These residual few mill watts of power are expected to emerge mainly from the splice reflections and Rayleigh scattered components of the high-power clockwise direction. At this stage the total output power is located in the clockwise direction only and therefore the ring is said to be lasing unidirectionally. This unidirectionality is sustained up to the maximum pump power value of 18 W and from there onward for decreasing pump powers down to the lower switching point. Here, the powers from both directions jump back to similar values, and ring cavity starts to emit again bidirectionally. The hysteresis region prevalent in Fig. 2a is also shown in the individual output powers.

The switching behavior at the upper as well as the lower switching point is sudden up to the accuracy of 60 mW pump power, the maximum resolution of pump power supply we used. The behavior was reproducible over many weeks with only slight changes in the values of switching points < 0.5 W, which presumably originate due to changes in the environmental conditions.

**Figure 3:**
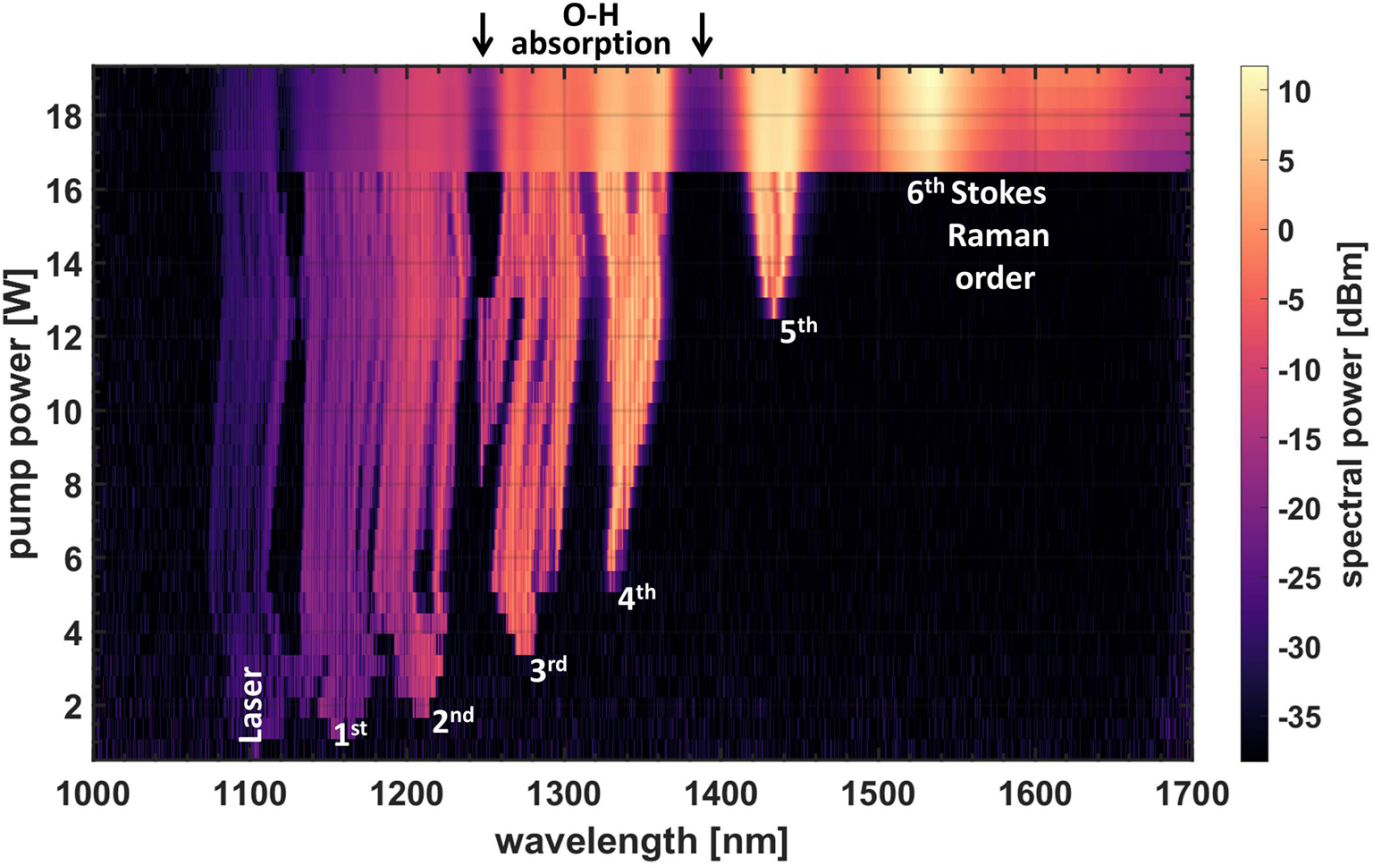
General example for the spectral evolution of the final direction output during increase of pump power starting at the laser wavelength around 1100 nm and broadening by 6 Stokes Raman orders and additional wavelength components up to 1700 nm.

The spectral evolution of the final direction during increase of pump power is displayed in Fig. 3. The optical power is not confined to the laser wavelength only, but due to the high-power density in combination with the virtually infinite propagation distance of the ring cavity a broad continuum of wavelengths is excited. This makes this approach also relevant to the scientific community regarding supercontinuum generation due to its uncommon approach of generating a broad wavelength spectrum. Especially where a continues-wave supercontinuum is to be generated at low power levels, the ring cavity’s self-seeding nature of Stokes Raman scattering strongly facilitates this goal. In addition, no special fibers for dispersion management are required. It is a simple and easy self-made continuous wave supercontinuum source already way below the power and length requirements of the DI threshold. In addition, this concept can be transferred straight forward to other pump wavelengths.

Prior to the pump power of the upper switching point up to 5 Stokes Raman orders broadened by additional nonlinear effects are visible, superimposed by a complex spectral fine structure. With the nonlinear switching to unidirectionality a 6th Stokes peak appears accompanied by a broad background up to the analyzer’s wavelengths limit. Interestingly, the complex fine structure has vanished, and the entire spectrum now has a very smooth envelope. The spectral gaps which remain around 1250 nm and 1380 nm originate from elevated propagation losses in the km long passive fiber due to O–H absorption.

Above the upper switching point we observe a similar spectrally broadened output in both directions albeit the final direction holds two orders of magnitude more power. This is a clear indication that power and spectrum of the disabled direction originate from the final one, e.g. due to reflections on internal splices or due to Rayleigh scattering. The power in the disabled direction is too low to generate a similar spectral broadening.

For the spectral broadening we expect the main contributing effect to be the Raman Effect starting at the laser peak around 1100 nm and progressing to longer ones. We suppose that these longer components can be simply be accounted for as (nonlinear) loss for the laser wavelength in addition to other (linear) loss sources like fiber attenuation or the output coupler and that detailed information about e.g. the number of excited Stokes Raman orders or the spectral fine structure are not important to describe and understand the origin of the DI. This assumption would be invalid if there are additional nonlinear effects involved that transfer energy back from longer wavelength to the laser peak. There are several nonlinear effects that in principle transfer energy to smaller wavelength. Parametric effects like four wave mixing can do this but they all require phase matching to be efficient. Such a phase matching requires a special dispersion profile not available in the single mode range of standard fibers like Corning HI-1060 used here. Another effect might be dispersive wave generation in combination with soliton fission during supercontinuum generation in the anomalous dispersion range. Such solitons are not apparent in the spectrum which excludes this effect.

### Impact of length and loss on the upper switching point

**Figure 4:**
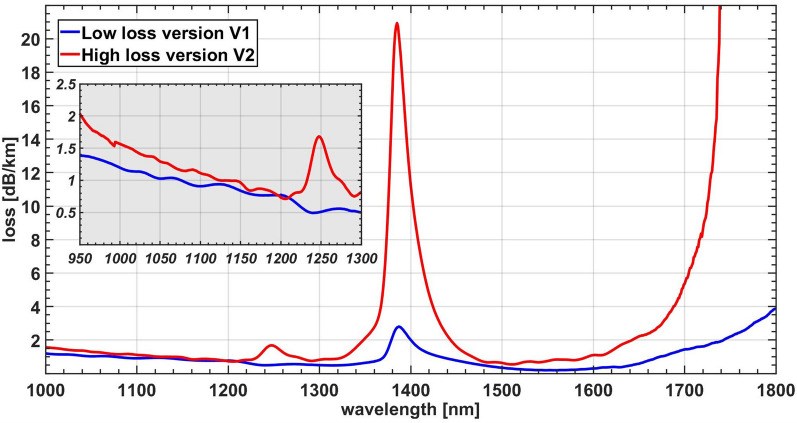
Attenuation as a function of wavelength for the two versions of Corning’s HI1060 fiber. The inset highlights the region of the laser wavelength.

To influence the upper switching point we composed the passive fiber section by 1 km long sections of two different fibers. Both fibers are Cornings HI-1060, share the same data sheet, and meet its requirements at the design wavelength of 1060 nm. Nevertheless, the spectral dependence is significantly different as shown in Fig. 4. The loss peaks in the spectrum are typical resonances of O–H bands. Hence, extra effort was expended for the low loss version V1 to remove water. We had one km available from the low loss version V1 and three kilometers from the high loss version V2.

So far, we do not know whether only the linear loss at the laser wavelength is influencing the phenomenon. Due to the broad spectrum involved, it is conceivable that also the losses at other wavelengths, especially the high loss water absorption peaks, are influencing the nonlinear loss experienced by the laser wavelength through changes in the rate of simulated Raman scattering.

**Figure 5:**
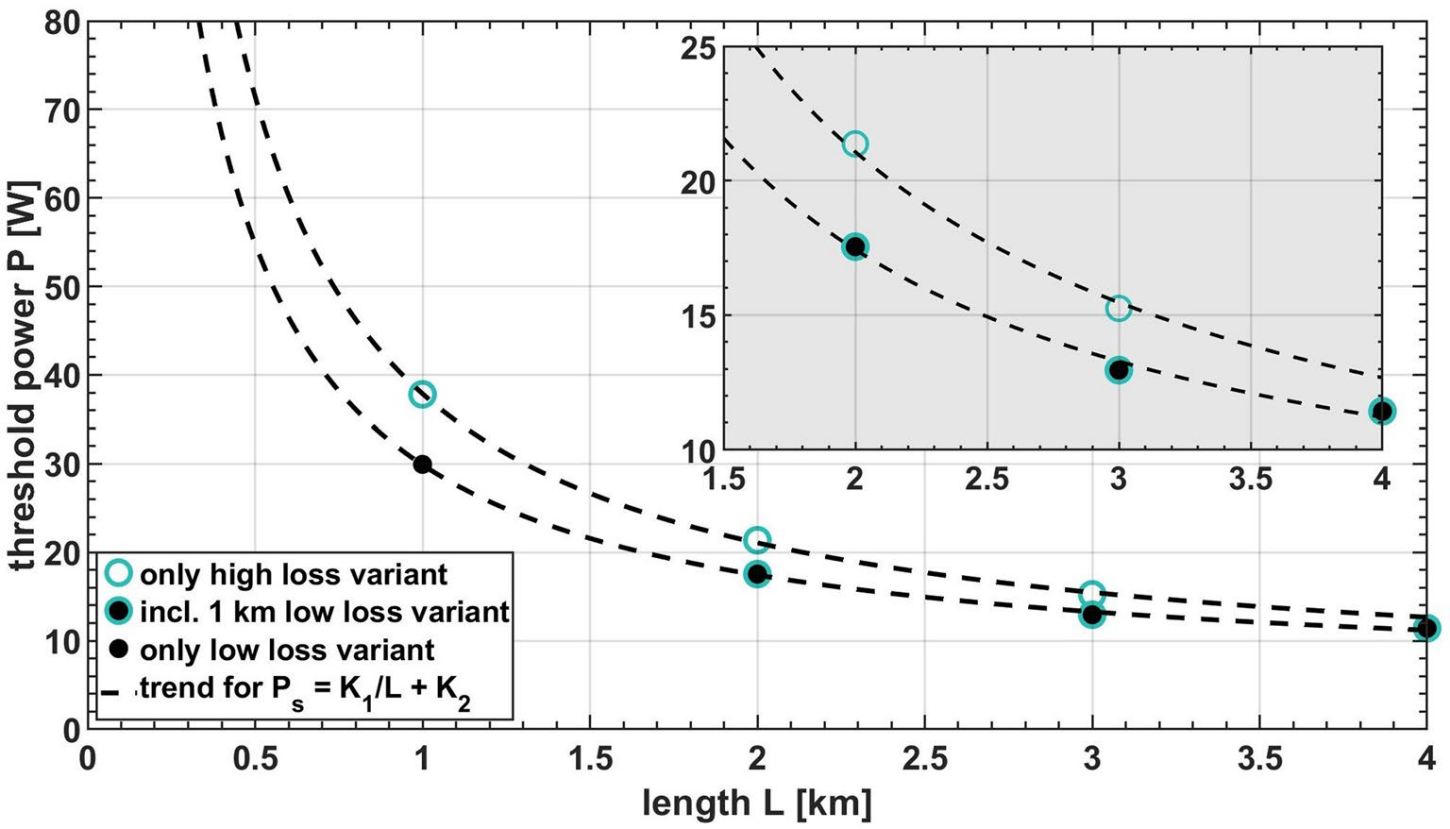
DI threshold power as a function of passive fiber length and loss. The inset highlights the power difference between two trends.

By varying fiber length and loss we investigated their impact on the upper switching point with respect to the pump power. Here, the threshold power is defined as the highest pump power before the systems switches to unidirectional lasing. The results are shown in Fig. 5.

**Figure 6:**
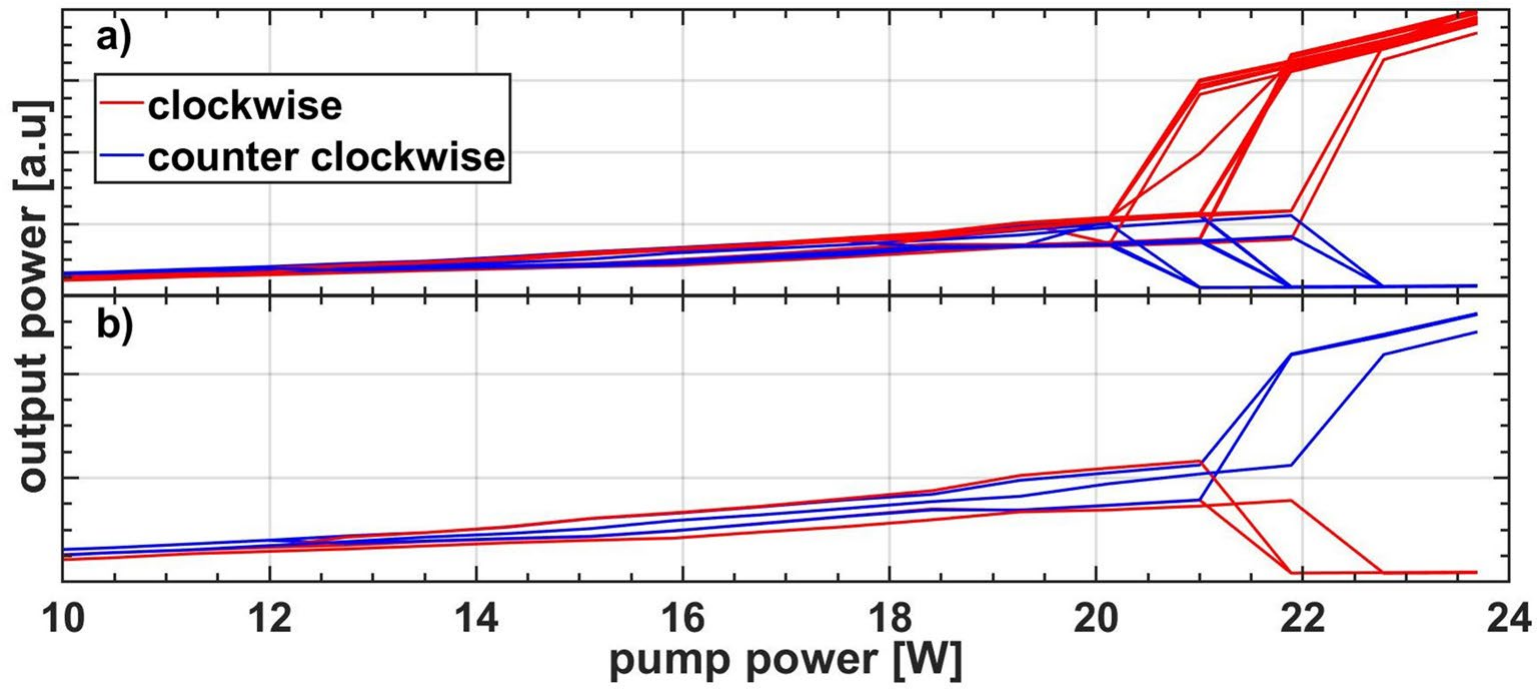
Output power as a function of increasing pump power separated in runs where the cavity switched to (**a**) the clockwise and (**b**) the counterclockwise direction. There are 30 runs in total. The cavity contained 2 km high loss fiber V2.

The highest power is required for the shortest cavity comprising the high-loss fiber only. Increasing the passive fiber length reduces the DI threshold, and so does the inclusion of the low-loss fiber. Generally, nonlinear effects scale with power times length and corresponding trends are included in Fig. 6, separated for both cavity versions, with and without the low loss fiber V1, respectively. The data points resemble the trends quite well, indicating a nonlinear origin for the DI phenomenon. Regarding a minimized passive fiber length, values down to approx. 0.3 km seem currently reasonable. A further reduction is limited by the steep slope of the power-times-length-trend for shorter fiber lengths.

Additionally, the data also clearly shows that a lower fiber loss reduces the threshold power. Note that so far we do not know at which wavelength region the loss difference matters, probably within the entire region covered by the spectrum. At the laser wavelength of 1100 nm the linear loss of both fiber versions differ by 0.2 dB/km only, or mere 5%/km. In contrast the trends for the threshold power differ by 12% at 4 km up to 22% at 1 km. Total fiber losses are already quite low in fiber V1 and we see only minor possibilities for optimization here. But especially for short fiber lengths < 0.5 km where the slope of the power times length scaling is quite steep also minor improvements might result in significant reduction of the power or length requirement. As reference the two trends depicted in Fig. 5 suggest at 80 W pump power a fiber length of 450 m for the high loss version and of 350 m for the low loss version.

The loss at other wavelengths can influence the extent of the broadening due to stimulated Raman scattering, thereby influencing the nonlinear loss observed by the laser wavelength and in consequence impacting the DI threshold power. If this connection turns out to be true, an interesting possibility to influence the DI threshold would be to introduce a high linear loss already in the region of the first to second Raman Stokes peak. This can be implemented with a strong wavelength dependent coupling ratio found e.g. in couplers for wavelength division multiplexing. Currently we think that the particular nonlinear effect (like Raman scattering) is not important but that the phenomenon is about nonlinear loss of the laser wavelength in general and that any kind of nonlinear loss can serve the purpose and trigger DI. Something similar is known for the phenomenon of modulation instability where the continuous wave emission of a laser transitions into pulses beyond a certain pump level. This phenomenon requires a rather lossy cavity to occur, known as the bad-cavity condition, and it is not important how these losses are implemented (e.g. local or distributed, internal or through output coupling)^1^.

### States and sequences of operation

We stress, that the final direction is not predefined. The cavity can finally switch in any of the two directions. This observation is a clear indication, that we are dealing with an instability phenomenon and has an important impact on the investigation strategy. Obviously, uncovering this phenomenon is not about explaining why one direction is superior over the other, as for instance for the theta cavity design where the resulting propagation direction experiences less roundtrip loss than the other^19^. In our case with no predefined outcome, we are essentially looking for two explanations. The first explanation regarding unidirectional lasing is about the cause of the destabilization of the bidirectional lasing state with respect to pump or laser power. This must be distinguished from the second explanation regarding the choice of the final direction.

In Fig. 6 we present the results of a sample of 30 runs for the setup containing *2* km of the high loss fiber V2. During each run the output power in both directions was monitored simultaneously, while the pump power was incremented in steps of approximately 0.5 W up to the maximum diode power of 23.7 W. After this the power was decreased in the same manner down to where both output powers converged to a similar value again (not shown). The clockwise direction was chosen 27 times (90%) and counterclockwise direction 3 times (10%). The upper switching point or DI threshold is in the interval between 20 and 22 W.

**Figure 7:**
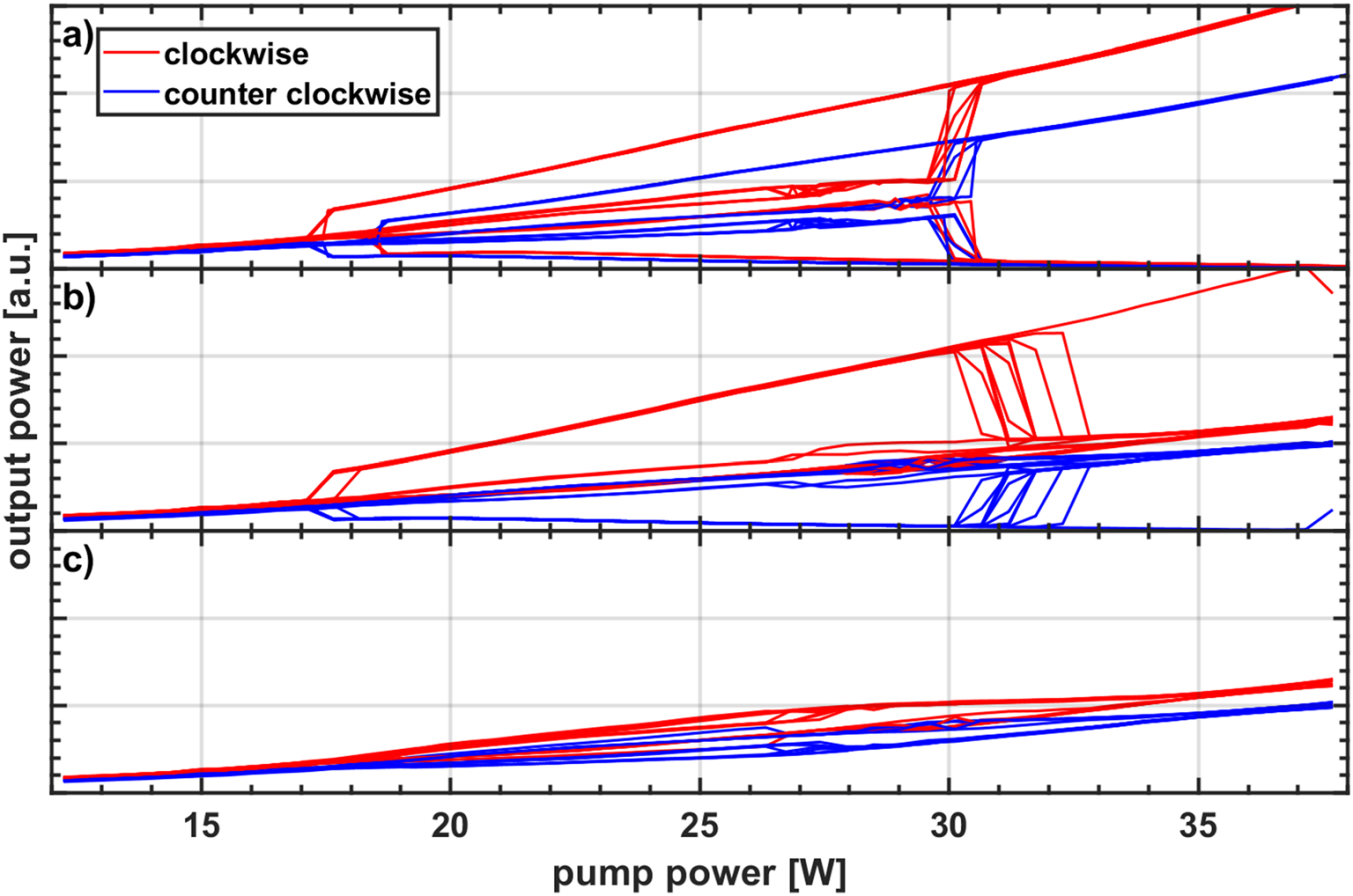
Superimposed output power traces for 30 power cycles of the cavity containing 1 km low loss fiber separated in switching events during (**a**) increase and (**b**) decrease of the pump power, or (**c**) without switching to unidirectional lasing.

A similar analysis was done for a 1 km long cavity with low loss fiber V1. The results are presented in Fig. 7. Regarding the final direction the laser switched 21 times (70%) into the clockwise direction and 3 times (10%) into the counterclockwise direction. It did not choose a final direction 6 times (20%). This contrasts with the longer cavities (2, 3, and 4 km), where a final direction was reliably observed whenever the threshold power was reached.

Another new observation compared to longer cavities is a retarded switch, meaning that the cavity did not switch to a single direction during increase of pump power but did so later at the downward branch of the power cycle. The specific distribution of switching events is shown in Table 1. Most of the times (47%) the cavity chose a final direction while the pump power was being decreased. The DI happened less frequently (33%) when the pump power was increased. This might indicate that the phenomenon is favorably triggered by a decreasing pump power. Additionally, during a retarded switch the cavity consistently preferred the counterclockwise direction. The causes for these behaviors are currently under investigation. Once understood they might be used to predefine the final direction.

**Table 1:** Distribution of switching events for the 1 km long cavity.

	Clockwise	Counterclockwise
Increase	7	3
Decrease	14	0

Out of 30 pump power cycles the cavity did not switch to a single direction 6 times.

96% of the switching events happened in a tight pump power range between 30 and 31 W albeit up to 37 W maximum pump power was used. Specifically, the events were not equally distributed in the range above the threshold. Most switching events were observed after the maximum pump power of 37 W was applied but no final direction was chosen until a lower pump power in the range of 31 W to 30 W was reached. This is the same region where the switching events happen during increase of the pump power. It appears, at least for the 1 km long cavity, that DI requires a quite specific pump power to occur and that it is not about a threshold pump power where any higher pump power is equally suited.

**Figure 8:**
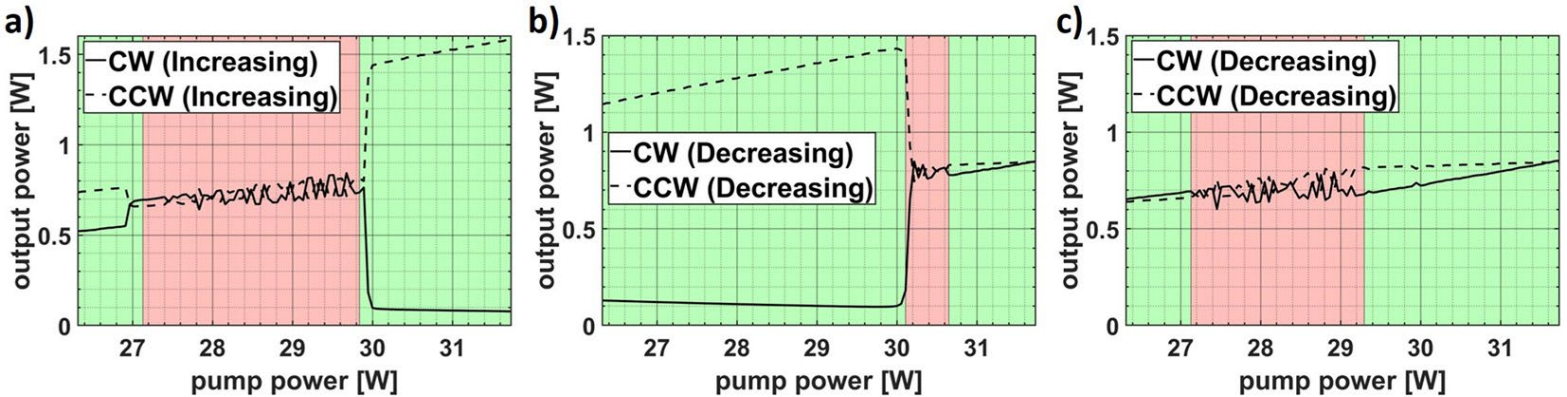
Detailed plots regarding the output power behavior for switching events happening (**a**) during power increase, (**b**) during power decrease, or (**c**) not at all. Regions with a fluctuating power trend are highlighted in red and with smooth trend in green.

A more detailed view on the output power in the vicinity of the upper switching point is presented in Fig. 8 for three selected examples (switch during power increase, switch during power decrease and a power cycle without switch). In addition, the power output is classified in two categories: smooth (highlighted in green) and fluctuating (highlighted in red).

Figure 8a shows that till around 27 W the output powers from both directions exhibit a smooth trend. For higher diode current values the output power trend starts to fluctuate. This fluctuating behavior is sustained till around 30 W. At this point the laser chooses a single direction and starts to run smoothly again.

A similar trend is observed in the case when the laser undergoes a retarded switch. As shown in Fig. 8b while decreasing the pump power the laser runs smoothly till around 30.5 W where the fluctuations begin. At 30 W the laser switches to a single direction and runs smoothly thereafter.

In Fig. 8c the smooth and fluctuating regions are also distinguished. But compared to the first two cases the laser runs smooth in the threshold region around 30 W. This might indicate that a certain amount of instability shown by the fluctuating output power trend is necessary and needs to be present in the threshold region to trigger DI.

Note that the fluctuations correspond to the single direction powers only. With similar magnitude but opposite sign they cancel each other out and are not visible with respect to the total emitted power of the cavity. It is neither the pump power nor the total emitted power that fluctuates.

## Summary and conclusion

With this manuscript we introduced the phenomenon of direction instability (DI). Various cavity layouts where investigated to reveal some interesting facts about this instability.

Investigations concerning the dependence of the length and linear loss of the passive fiber on the DI threshold revealed a power times length relation. This indicates nonlinear optical effects to be the very nature of the DI phenomenon. The shortest realized cavity length was 1 km which requires approx. 30 W of pump power to trigger DI. Investigations of multiple DI instances revealed that the final direction is not predefined. While the clockwise direction is currently preferred in our setup, the counterclockwise direction nevertheless occurs with a probability of approx. 10%.

The alternating fluctuations shown in Fig. 8 signify a coupling of the two directions. By which effect (or effects) the two directions are coupled is yet to be clarified. With km long fiber lengths, an obvious origin of such directional coupling could be Rayleigh or Brillouin scattering, But the former is a linear phenomenon and the latter is only efficient for very narrow linewidths.

Another candidate, which is used in random fiber lasers, could be a population inversion dynamical grating forming in the active fiber section. As demonstrated in^17^, a ~ 0.1% reflection (easily obtained at a splice) can produce a 7% reflection inversion population dynamical grating which couples the directions. Rayleigh scattering in combination with stimulated Raman scattering is also employed for directional coupling in random lasers^14^ and could play a role for DI, as well, due to the similar long-length high-power cavity architecture.

As explained in^20^, the feedback caused by the disordered distributed Rayleigh scattering can be modeled as an ensemble of many Fabry Perot cavities, each with a different resonance. This results in spectrally narrow resonances, which are amplified initially by Raman scattering and are dense enough to fall into the Brillouin gain profile. Therefore, stochastic spikes were observed in the spectrum shown in^14^ at low powers signifying the presence of Brillouin scattering. At higher pump power spectral smoothening occurs, caused by additional nonlinear effects, one option being nonlinear linewidth broadening and thereby reduced efficiency of Brillouin scattering. We also observe a strong spectral smoothing when DI is triggered (Fig. 3), which might indicate a similar complex physical background.

With its unique approach of a single direction emission in a reciprocal ring laser it bears the potential for numerous applications. It might be an interesting alternative to existing approaches of enforced unidirectionality in ring lasers, especially suitable for high power applications or for new wavelength regions, where special components like an optical isolator do not exist.

The most important step towards application is a deterministic direction the cavity finally switches to. Fortunately, there already is a strong preference towards one direction and future work will mainly focus on the underlying causes and how to strengthen them even more. Another task is to ease accessibility by finding ways to lower the power and length requirements.
